# A novel model for evaluating thrombolytic therapy in dogs with ST-elevation myocardial infarction

**DOI:** 10.1186/s12872-016-0194-6

**Published:** 2016-01-25

**Authors:** Hong Zhang, Yong-chun Cui, Yi Tian, Wei-min Yuan, Jian-zhong Yang, Peng Peng, Kai Li, Xiao-peng Liu, Dong Zhang, Ai-li Wu, Zhou Zhou, Yue Tang

**Affiliations:** Animal Experiment Center & Beijing Key Laboratory of Pre-clinical Research and Evaluation for Cardiovascular Implant Materials, Beijing, 100037 People’s Republic of China; Center of Clinical Laboratory, State Key Laboratory of Cardiovascular Disease, Fu Wai Hospital, National Center for Cardiovascular Diseases, Chinese Academy of Medical Sciences and Peking Union Medical College, Beijing, 100037 China

**Keywords:** Thrombolytic therapy, White thrombus, Coronary artery occlusion

## Abstract

**Background:**

There is still no standard large animal model for evaluating the effectiveness of potential thrombolytic therapies. Here, we aimed to develop a new beagle model with ST-elevation myocardial infarction (STEMI) by injecting autologous emboli with similar components of coronary thrombus.

**Methods:**

18 male beagles were included and divided into three groups: red embolus group (*n =* 6), white embolus group (*n =* 6) or white embolus + rt-PA group (*n =* 6). Autologous emboli were infused into the mid-distal region of the left anterior descending coronary artery. The composition of embolus was examined by scanning electron microscope (SEM). Coronary angiography was performed to verify the status of embolism. Myocardial infarct size was measured by 2, 3, 5- triphenyltetrazolium chloride (TTC) staining.

**Results:**

Red thrombus was characteristic of loose reticular structure of erythrocytes under SEM, while the white embolus had compacted structure that mainly consisted of a dense mass of fibrin. Coronary angiography showed the recanalization rate was 2/6 in the red embolus group versus 0/6 in the white embolus group in three hours after occlusion. Arrhythmia, resolution of ST-segment elevation and lower T wave on the electrocardiogram appeared in the red embolus group but not in the white embolus group. Another six dogs with white thrombi were treated with rt-PA. Five out of six dogs exhibited coronary recanalization after two hours of therapy, compared to zero dogs without rt-PA treatment. The size of myocardial infarction in rt-PA group reduced significantly compared with white embolus group using TTC staining method.

**Conclusions:**

The white embolism model was more convenient experimentally and had a higher uniformity, stability and success rate. The major innovation of our study is that we applied fibrin-rich white thrombi to establish beagle model possessing features of clinically observed coronary thrombi in time window of intravenous thrombolysis of STEMI. This model can be used to evaluate new thrombolytic drugs for the treatment of STEMI.

## Background

Thrombolytic therapies are critical in salvaging ST-segment elevation (STEMI), which accounted for 25 % to 40 % of cases in myocardial infarction [1]. When percutaneous coronary intervention (PCI) cannot be administered in a timely manner [anticipated first medical contact (FMC) to device time > 120 min], thrombolytic therapies were recommended for STEMI according to the most recent ACCF/AHA and ESC guidelines [1, 2]. Pre-hospital fibrinolysis is an important intervention to salvage ischemic myocardium, improve prognosis and offer additional time for clinical treatment [3]. Most patients can benefit from thrombolysis, however, the specificity, effectiveness and safety of thrombolytic drugs are still required to be improved [4, 5]. To develop new drugs with faster effects and fewer side effects, it is essential to establish an animal model of the coronary artery embolism mimicking clinical status, especially the thrombus composition, good uniformity and repeatability. However, the coronary thrombi in previous animal models mostly were red or mixed emboli, which were different from that of clinical settings. The composition of the coronary thrombi in time window of thrombolysis was not clarified until the coronary thrombus suction technique was used. Recently, we understood the coronary thrombi in STEMI patients are mainly composed of fibrin with a small portion of platelets that decrease over time, a few erythrocytes, cholesterol crystals and leukocytes [6]. This kind of fibrin-rich thrombi is similar to those in cerebrovascular thrombosis. Kirchhof et al. used white embolus to make rabbit model of cerebral embolism to evaluate thrombolytic drugs [7], which has not been applied to the heart. The aim of present work was to set up an ideal arterial thrombus model that reflected the clinical syndrome in patients with STEMI. Therefore, we compared red and white embolism models via catheter injection into coronary arteries in animal models and investigated the effectiveness of thrombolytic drugs.

## Methods

### Animals

Twenty-one adult male beagles (12 to 17 kg) were used in this study, which was approved by the animal welfare and the ethical review committee of Fuwai Hospital, Chinese Academy of Medical Sciences (permission number 2013-2-30-BJK02). The animal procedures of this experiment were performed according to the guidelines from Directive 2010/63/EU of the European Parliament on the protection of animals used for scientific purposes.

### Preparation of emboli

Based on previous methods [7], we do some modification. Four hours before the operation, 3 ml autologous venous blood was collected from each experimental animal to prepare the individual matched emboli. For white embolus: the venous blood without anticoagulant was centrifuged at 1500 rpm, for 5 min at 4 °C. After extracting the supernatant and injecting it into a silicone tube (2.5 mm in diameter), the blood clots were put into a 37 °C water bath for 0.5 h. The blood clots were extruded into a sterile plate by needle tubing for automatic retraction for 3 h (about 1.2 mm in diameter) and were cut into 5 mm long cylinders. For the red embolus: the blood in the syringe was directly injected into a silicone tube, followed by incubation at 37 °C for 0.5 h. The subsequent processes were identical to those used to make the white embolus. We also measured the concentrations of fibrin, platelets and erythrocytes in whole blood or supernatant. Fibrin parameters were determined by an automatic coagulation analyzer (STA-Revolution, Stago), platelets and erythrocytes were measured by an automated hematology analyzer (XE-2100, Sysmex).

### Coronary artery thrombosis embolism model

Animals were anesthesied with ketamine (35 mg/kg) and diazepam (15 mg/kg) and maintained with the same drugs (dose = 1/2 of induction) administered once every hour. Fentanyl (0.03 mg/kg) was used for analgesia during the operation and post-operation. After anesthesia, animals were affixed to the operation table in the supine position and given endotracheal intubation for assisted respiration in synchronized intermittent mandatory ventilation (SIMV) mode (Savina, Draeger Medical AG&Co.KG, Germany). The parameters include tidal volume (10 ml/kg), breathing rate (20 times/min), expiration/inspiration (E/I) ratio (1:1.5–2) and oxygen saturation (55 %). The arrhythmias were also monitored (M8005A, Philips Medizin Systeme Boeblingen GmbH, Germany). The branchiocephalic vein of the left forelimb was used for heparin and rt-PA injection. An artery sheath catheter was inserted in the axillary artery branch of right forelimb. Under fluroscopic guidance of C arm X-ray (9800–12, Beijing Tongyong Medical Equipment Co., Ltd, China), a 5 F catheter was inserted into the left coronary artery to obtain a coronary angiogram. Keeping the 5 F catheter positioned at the left anterior descending artery near the first diagonal branch, one embolus were injected to occlude blood flow through the mid-distal region of the LAD. Because the catheter cannot be inserted too deeply, the embolus might reach the diagonal branch. So, the operator must carefully handle and avoid the embolus flowing into the vessels with lower pressure.

### Thrombolysis treatment

After LAD occlusion for 60 min, 1000 U heparin sodium injection was henceforth given once every two hours. The rt-PA infusion (0.4 mg/kg) was given as a loading dose, and the thrombolytic agent was continuously infused over 30 min (1.2 mg/kg) afterwards. The remainder of the rt-PA was continuously infused over 60 min (0.8 mg/kg). This protocol was according with drug specification and the dose used in dogs was based on the equivalence of clinical safe dosage [8].

### Measurements of coronary perfusion

The animals received electrocardiogram (ECG) examination before embolus injection, at the moment of injection and every 15 min for three hours after injection to record the changes of ST-segment, T wave and other variations to estimate the statuses of embolism. For coronary angiogram, animals received right anterior oblique, anteroposterior and left anterior oblique coronary angiography before injection of the embolus, at the moment of injection and every 30 min for three hours after injection to evaluate the degree of occlusion and/or autolysis. Coronary angiogram was also carried out at 10, 20, 30, 60, 90 and 120 min after using rt-PA or at the time of occurrence of arrhythmia or electrocardiographic changes to evaluate the thrombolytic effects. Reperfusion time was defined as the time when recanalization was verified by coronary angiogram [9].

### Pathological studies

Autologous emboli were analyzed by scanning electron microscope (SEM). After three hours automatic retraction, specimens were washed three times with phosphate buffer, fixed for 120 min in 2 % glutaraldehyde and rinsed three times with phosphate buffer. Samples were then fixed for 120 min with osmic acid, rinsed, and dehydrated in a graded series of ethanol concentrations (50 %, 70 %, 90 % and 100 %) over a period of 40 min and further dehydrated in a graded series of concentrations (50 %, 70 %, 90 %, 100 %) of isoamyl acetate-ethanol solvent. The clots were dried with hexamethyldisilazane for 10 min and fractured naturally through pulling to obtain a fracture surface for analysis. Finally, clots were coated with gold-palladium prior to examination in a scanning electron microscope (TM-1000, HITACHI).

The infarct size was determined by 2,3,5-triphenyltetrazolium chloride (TTC) staining. The animals were anesthetized by intravenously injecting Ketamine (35 mg/kg) combined with diazepam (1.5 mg/kg) and euthanized through injecting 10 % potassium chloride (15-20 ml) after that. The hearts were excised and cut cross-sectionally into plates with 10 mm thick and stained with 2,3,5-triphenyltetrazolium chloride (TTC) [10]. The infarct size was identified as the non-TTC-stained area and the infarct ratio (%) is the ratio of area of infarction size to area of left ventricular. We also dissected LAD to observe the situation of thrombolysis. The skin, mucosa, hearts, brains, lungs, livers, spleens and kidneys were subjected for microscopic examination to estimate the bleeding risk according to the previous study [11].

#### Statistical analysis

Data were analyzed by SPSS 10.0 software (Chicago, IL: SPSS Inc.) and presented as mean ± SEM. The frequency of recanalization was statistically analysed with Fisher’s exact, 2-tailed test. Infarct size ratio was evaluated by unpaired Student’s *t*-Test. *P* < 0.05 indicated a significant difference.

## Results

A total of twenty-one dogs were used in the experiment. One animal died of ventricular fibrillation due to the extended duration of the catheter in the LAD. Two animals had diagonal branch embolisms. These three animals were excluded from the study. Finally, eighteen animals were divided into three groups, including red embolus group (*n =* 6), white embolus group (*n =* 6) and white embolus + rt-PA group (*n =* 6).

### ECG

**Fig. 1:**
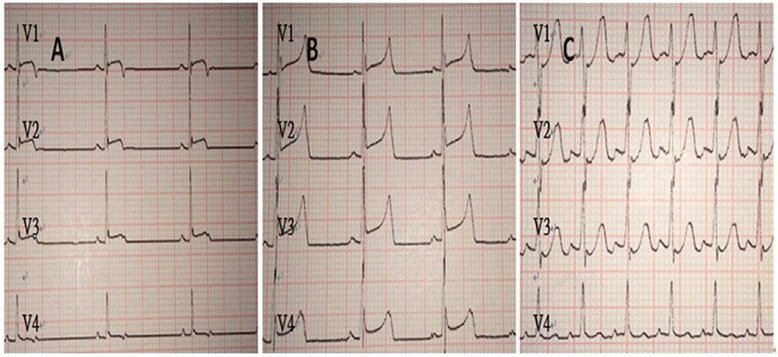
Example of lead V1-V4 of white embolus + rt-PA group. **a** Baseline ECG. **b** Acute ECG injury pattern of LAD occlusion following white embolus injection, exhibiting marked ST segment elevation. **c** ECG of the dog after using rt-PA; note the relative normalization of the ST segment and elevated heart rate

The ECG of all animals was normal before the operation. Transient premature ventricular fibrillation occurred in two cases in the red embolus group and four cases in the rt-PA group and was reversed to sinus rhythm after defibrillation. Furthermore, these six animals were present coronary recanalization (reperfusion) according to coronary angiogram as described below. ST-segment and T wave of lead V1-V4 elevated after injection of autologous emboli. ST-segment resolution and lower T wave appeared on the ECG in the red embolus group at 120 min, indicating that the embolism autolyzed. Neither of these was observed in the white embolus group. ST-segment resolution and a lower T wave were observed after the administration of rt-PA to dissolve the white embolus (Fig. 1).

### Coronary angiogram

**Fig. 2:**
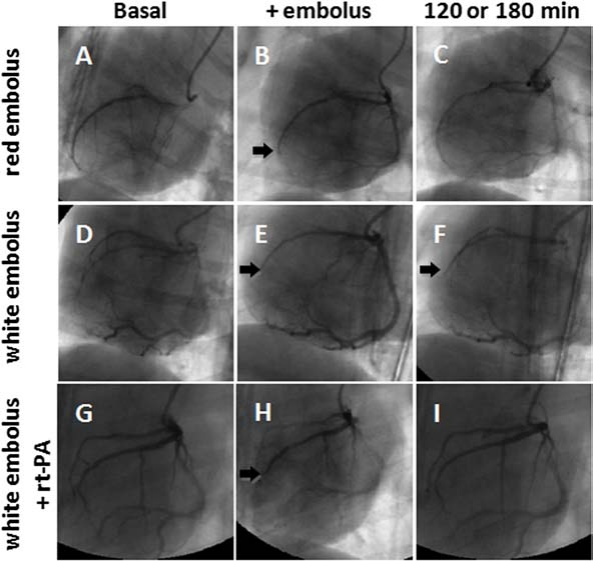
Coronary embolism changes at different time points were revealed by coronary angiography. The up-panel images represented respectively: before (**a**), 60 min (**b**) or 120 min (**c**) after occlusion with red embolus; mid-panel images represented respectively before (**d**), 60 min (**e**) or 180 min (**f**) after occlusion with white embolus; bottom-panel images represented respectively before occlusion (**g**) by white embolus in the rt-PA group; 60 min after injection of the white embolus (**h**) and 120 min after rt-PA treatment (**i**). Arrows in (**b**), (**e**), (**f**) and (**h**) indicate the location of embolus

The preoperative coronary angiograms showed that all coronary arteries in the experimental animals were normal. Three hours after LAD occlusion, two animals in red embolus group appeared to have recanalization and the embolism position in white embolus group was still in the mid-distal LAD. These results indicated the white embolus might be better for subsequent experiments. In addition, five out of six animals (5/6) received rt-PA had recanalization flow compared with none in control group (0/6; *p =* 0.015) after two hours (Fig. 2). The average time to reperfusion was 43.2 ± 7.4 min in the rt-PA group (Table 1).

**Table 1:** Effects of rt-PA on thrombolysis

	rt-PA group	white embolus group	P value
Thrombolytic effect			
Reperfusion rate	5/6	0/6	0.015
Reperfusion time (min)	43.2 ± 7.4	--	--

In the rt-PA group, coronary recanalization was achieved in 5 of 6 animals. The reperfusion time was 43.2 ± 7.4 min. There was no coronary recanalization observed in the white embolus group

### Pathological studies

**Fig. 3:**
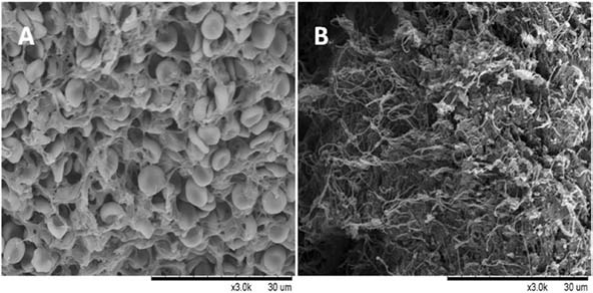
The characteristics of different thrombi were evaluated by SEM. **a** The structure of whole blood thrombus was incompact and sparse cellulose meshes were full of erythrocytes. **b** White thrombus with compacted structure that mainly consisted of a dense mass of fibrin

SEM examination was carried out to identify the characteristics of different autologous emboli (Fig. 3). Blood parameters of whole blood and supernatant after emboli preparation were shown in Table 2. White emboli were more rigid than whole blood emboli because the white emboli contained more fibrin and less erythrocytes under same volume.

**Table 2:** Blood parameters of whole blood and supernatant for emboli preparation

Blood parameters	Mean ± SD	n
Erythrocytes in whole blood, ×10^12^/L	7.45 ± 0.37	12
Platelets in whole blood, ×10^9^/L	319.00 ± 45.80	12
Platelets in supernatant^a^, ×10^9^/L	456.00 ± 51.50^*^	6
Fibrinogen in whole blood, g/L	2.21 ± 0.04	6
Fibrinogen in supernatant^a^, g/L	4.09 ± 0.25^*^	6

^a^The supernatant was extracted from the venous blood and centrifuged at 4 °C and 1500 rpm for 5 min

^*^ *p* < 0.05 compare with whole blood

After autopsy, no obvious signs of bleeding were seen in the skin, mucosa, heart, brain, lungs, liver, spleen, kidneys or other important organs (data not shown).

**Fig. 4:**
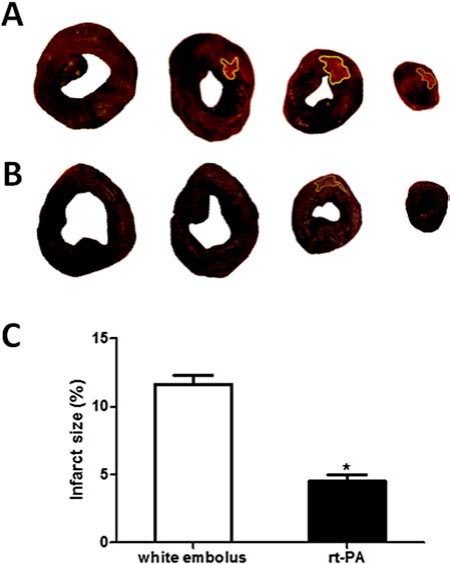
Infarct size ratio. **a** Myocardium stained with 2,3,5-triphenyltetrazolium chloride(TTC) in white white emboli group. **b** Myocardium stained with TTC in rt-PA group. **c** The infarct size of rt-PA group and white emboli group are 1.1 ± 0.19 cm^2^ and 2.86 ± 0.18 cm^2^, respectively. The infarct size ratio (%) in rt-PA group was significantly smaller than it of white emboli group (4.48 ± 0.52 vs. 11.61 ± 0.64, *p < 0.01). ▇ = rt-PA group (*n =* 6), □ = white emboli group (*n =* 6) Vertical bars represent SEM

For infarct size measurement, individual slices were photographed in color using Image J, and the extent of myocardial necrosis was determined by quantifying the unstained sections of the heart. Infarct size ratios were 11.61 ± 0.64 % and 4.48 ± 0.52 % in the white embolus group and rt-PA group respectively (Fig. 4). The situations of thrombolysis detected through coronary artery dissection were in accordance with the results of coronary angiogram.

## Discussion

About 70 % of cases of acute coronary thrombosis are associated with a disrupted atherosclerotic plaque and about 30 % of them are only with superficial intimal injury [12]. In case of plaque rupture or endothelial damage, the exposure of collagen and tissue factor triggers the activation of platelets and coagulation factors, which result in thrombus formation [13, 14]. The different components of thrombus in coronary artery can affect the pathological process of STEMI and the thrombolytic effect directly. In recent years, the pathological analysis of aspirated intracoronary thrombi demonstrated that about 65 % of patients had platelet-rich (white) thrombi, particularly in the early hours of AMI, the remaining 35 % of cases had erythrocyte-rich (red) thrombi with low thrombolysis in myocardial infarction (TIMI) flow [15]. Silvain et al. found that intracoronary thrombi were mainly composed of fibrin with the median ischemic time of 175 min. The fibrin content increased with the ischemic time, whereas the platelet content decreased and the erythrocyte content had no changes [6]. The results showed that there were fibrin-rich thrombi in the early time (<3 h) after onset of STEMI. The white thrombi in our experiments are similar to the fibrin-rich thrombi in patients with STEMI. Erythrocytes may contribute more to thrombus composition at later stages but not in the time window of acute reperfusion of STEMI. Red thrombus are composed of fewer massed platelets and more erythrocytes [16]. As previously reported, the ratio of erythrocytes determines the size of the pores between cellulose meshes. The softer thrombi with larger pores [7] are easier to be penetrated by fibrinogenase to dissolve. In this study, after injecting of red thrombi into coronary artery of dogs, the fibrinolytic system was activated, and the thrombi started dissolved through coronary angiogram, ECG and occurrence of arrhythmia. Whereas white thrombus with compacted structure has strong ability to resist fibrinolysis and no thrombolytic phenomenon occurred obviously. Recent experiments have reported the high stability of white emboli used in animal models in agreement with our observations [7, 17]. Overall, the white thrombus is more stable in animal model and has similar components of STEMI patients in early time of onset of this disease. These results made the animal model suitable for subsequent application in evaluating thrombolytic therapy.

The new model has better uniformity not only in embolus preparation but also in animal model establishment. Firstly, the white thrombi have identical components. To mimic this kind of fibrin-rich white thrombus, centrifugation conditions of 1500 rpm at 4 °C for 5 min were chosen. The blood parameters, mainly including platelet and fibrinogen, in the supernatant are at the similar levels. To avoid affecting the antifibrinolytic ability of thrombi, we did not use any anticoagulants in emboli preparation [18]. By operating quickly, we could guarantee that the supernatant did not coagulate before the silicone tube shaping step. From the SEM analysis, it was clear that white emboli with compacted structure mainly consist of a dense mass of fibrin. Secondly, the size, length and number of thrombi can be controlled and the locations of thrombi in coronary artery were similar. Through the guidance of catheter positioning, emboli were sent to anterior descending coronary artery and could be stuck in LAD with similar diameters. This model mimics the clinical situation that the fresh clot was broken off and carried through the flow into distal coronary artery at early stage of myocardial infarction. Thrombolytic therapy in myocardial infarction is a dynamic process due to the progressive embolus dissolution. In our experiment, we found the white embolus moved forward for a limited distance after using rt-PA during early stage, then it formed eccentrically clot in the distal section of coronary artery. The white embolus could be completely dissolved and LAD became recanalized in a certain time period.

The anti-fibrinolytic ability of the embolus is very important. Looser or more rigid thrombi do not behave like the endogenous thrombi in STEMI and therefore may not provide accurate information concerning the efficacy and safety of different thrombolytic drugs. There are no universal criteria to evaluate autologous emboli, and the different methods resulted in different experimental outcomes [18]. In current experiments, we used the autologous white embolism model to examine the thrombolytic actions of rt-PA. This drug is commonly used in clinical practice because of its rapid clearance and ability to be co-administered with heparin. The average time to reperfusion was 43.2 ± 7.4 min and the patency rate in this model was 5/6(83 %) in 90 min, which was similar to those in clinical study (73–84 %) [8, 19, 20]. The size ratio of myocardial infarct in rt-PA group reduced significantly compared to control group, which confirmed that use of thrombolytic drugs timely could improve the prognosis of patients with STEMI.

As determined by microscopic examination, there was no obvious bleeding-induced damage to skin, mucosa, heart, brain, lungs, liver, spleen, kidneys or other important organs. It may be related to the safer dose adopted in healthy animals. There is no perfect system to predict bleeding risk associated with thrombolysis in clinical treatment, so the animal experiments to evaluate bleeding risk is needed for further studies. Because the time window of thrombolytic treatment is relatively short [19, 21, 22], fast thrombolysis is very important for developing new thrombolytic agents. In conclusion, our model can be used to compare the rates and time of recanalization among different thrombolytic drugs in their safe dose ranges.

Catheter-based delivery of the autologous emboli was shown to be effective in our study. We selected the axillary artery branch instead of carotid artery or femoral artery as the puncture path for the first time, which could shorten the length of interventional devices in the body, reduce the risk of postoperative infection and decrease hemorrhage at the puncture point. In terms of detection, coronary angiography was effective at instantaneously ascertaining the degree of coronary artery stenosis, allowing the progress of thrombolysis to be carefully monitored. Because of its gentle temperament and homogenous genetic background, the beagle is widely used in preclinical drug evaluation. However, the diameter of coronary arteries in beagle is small and no specialized coronary artery catheters are currently available for these animals. Our protocol resolved this bottleneck problem.

At present, the methods for evaluating thrombolytic therapy in large animal models mainly include electrical injury [23], open chest thrombosis injection [24], balloon occlusion and thrombin injection [25] and copper coil-induced coronary thrombosis [26]. The first two methods require open chest operation, with complex operations and a larger trauma. The thrombus in the last two models may have different compositions from those seen in STEMI patients. Although the thrombus induced by electrical injury have similar composition to human coronary artery thrombi, this method takes a long time (3.2 ± 0.4 h) to complete coronary occlusion and the size of thrombus cannot be controlled [23]. The present model was a simple, efficient and inexpensive method with a smaller trauma. Furthermore, the artificially produced fibrin-rich white thrombus has uniform size and clinical features of coronary thrombi in time window of intravenous thrombolysis of STEMI.

## Conclusions

We established, for the first time to our knowledge, the coronary artery embolism model with white thrombus which had better stability, uniformity and a higher success rate. Importantly, the model produced thrombi with characteristics similar to those in STEMI patients in time window of thrombolytic therapy and was amenable to evaluation of thrombolytic therapies. This model can be used to evaluate new thrombolytic drugs for the treatment of STEMI.

## Abbreviations

ECG: Electrocardiogram
E/I: Expiration/inspiration
FMC: First medical contact
LAD: Left anterior descending artery
PCI: Percutaneous coronary intervention
rt-PA: Recombinant tissue plasminogen activator
SEM: Scanning electron microscope
SIMV: Synchronized intermittent mandatory ventilation
STEMI: ST-segment elevation
TIMI: thrombolysis in myocardial infarction
TTC: 2,3,5-triphenyltetrazolium chloride
